# Regulation of Myostatin on the Growth and Development of Skeletal Muscle

**DOI:** 10.3389/fcell.2021.785712

**Published:** 2021-12-24

**Authors:** Ming-Ming Chen, Yi-Ping Zhao, Yue Zhao, Shou-Long Deng, Kun Yu

**Affiliations:** ^1^ College of Animal Science and Technology, China Agricultural University, Beijing, China; ^2^ Tianjin Key Laboratory of Agricultural Animal Breeding and Healthy Husbandry, College of Animal Science and Veterinary Medicine, Tianjin Agricultural University, Tianjin, China; ^3^ NHC Key Laboratory of Human Disease Comparative Medicine, Institute of Laboratory Animal Sciences, Chinese Academy of Medical Sciences and Comparative Medicine Center, Peking Union Medical College, Beijing, China

**Keywords:** myostatin, skeletal muscle development, myogenesis, protein synthesis, degradation

## Abstract

Myostatin (MSTN), a member of the transforming growth factor-β superfamily, can negatively regulate the growth and development of skeletal muscle by autocrine or paracrine signaling. Mutation of the myostatin gene under artificial or natural conditions can lead to a significant increase in muscle quality and produce a double-muscle phenotype. Here, we review the similarities and differences between myostatin and other members of the transforming growth factor-β superfamily and the mechanisms of myostatin self-regulation. In addition, we focus extensively on the regulation of myostatin functions involved in myogenic differentiation, myofiber type conversion, and skeletal muscle protein synthesis and degradation. Also, we summarize the induction of reactive oxygen species generation and oxidative stress by myostatin in skeletal muscle. This review of recent insights into the function of myostatin will provide reference information for future studies of myostatin-regulated skeletal muscle formation and may have relevance to agricultural fields of study.

## Introduction

Myogenesis, the process of skeletal muscle formation, is a highly coordinated multistep biological process driven by many regulatory factors, such as paired box family proteins 3 and 7, myogenic regulatory factors (myogenin, MyoD, Myf5, and MRF4/6), and myocyte enhancer factor 2 family proteins. These factors collectively regulate the expression of muscle-specific genes and control skeletal muscle development (Braun and Gautel, 2011). Skeletal muscle satellite cells, which constitute the primary muscle stem cells, are located between the myofiber membrane and basal lamina (Sacco et al., 2008; Mashinchian et al., 2018). The activation, proliferation, migration, alignment, fusion, and differentiation of skeletal muscle satellite cells to form contractible, beating multinucleated myotubes are crucial for myogenesis (Yin et al., 2013; Zanou and Gailly, 2013; Hernandez-Torres et al., 2017).

Myostatin plays a key role in the development of skeletal muscle. After animals are born, the myostatin gene negatively regulates the growth and development of skeletal muscle by limiting the number and size of muscle fibers. Myostatin and its effect on meat yield have undergone intense study in the field of animal breeding. Myostatin is highly conserved in mammals, and loss-of-function mutations lead to an increase of skeletal muscle weight and produce a “double-muscle” phenotype has been reported for many species, including cattle, sheep, pigs, rabbits, and humans (Kambadur et al., 1997; Grisolia et al., 2009; Dilger et al., 2010). However, the molecular mechanisms of myostatin gene mutation and negative regulation of skeletal muscle growth and development remain controversial. Therefore, we focus here on the structure and function of myostatin, especially the similarities and differences between myostatin and structurally related members of the transforming growth factor-β (TGF-β) superfamily. In addition, we extensively summarize the regulatory effects of myostatin on myogenic differentiation, muscle fiber type transformation, and skeletal muscle protein synthesis and degradation. The role of myostatin in inducing reactive oxygen species (ROS) production and oxidative stress in skeletal muscle is also discussed. In short, we provide an update on recent insights into the function of myostatin, which may have relevance to agricultural fields of study.

## Myostatin and the TGF-β Superfamily

Myostatin, also known as growth differentiation factor-8 (GDF-8), is a member of the TGF-β superfamily and negatively regulates the growth and development of skeletal muscle through autocrine and paracrine signaling pathways (Gao et al., 2013). In mammals, the structure of the myostatin gene, *MSTN*, is relatively conservative, consisting of two introns and three exons, and it encodes 375 protein residues. The myostatin protein has characteristics typical of TGF-β family members, such as an N-terminal secretory signal peptide sequence, a protein hydrolysis site with an RSRR sequence, and a cysteine-rich C-terminal domain (McPherron and Lee, 1997). First, the precursor protein of myostatin, pro-myostatin, is synthesized. The myostatin precursor protein consists of three parts, namely the signal peptide, coding pre-peptide (N-terminal), and coding mature peptide (C-terminal) (Wolfman et al., 2003). The precursor protein is hydrolyzed twice to produce active biomolecules; the first cleavage occurs 24 residues from the N-terminus and is responsible for the removal of the signal peptide; the second takes place at the RSRR site to produce the C- and N-terminal polypeptides (Wolfman et al., 2003). Co-expression of pro-myostatin and latent-transforming growth factor beta-binding protein 3 (LTBP-3) sequesters pro-myostatin in the extracellular matrix, and secreted pro-myostatin can be cleaved extracellularly by the proprotein convertase furin (Anderson et al., 2008). Co-expression of LTBP-3 and myostatin reduces the phosphorylation of Smad2, and ectopic expression of LTBP-3 in mature mouse skeletal muscle increases fiber area, which is consistent with a reduction of myostatin activity (Anderson et al., 2008). Finally, the C-terminal peptide forms the mature disulfide-linked myostatin dimer, which is secreted into the bloodstream, where it plays its role (Wolfman et al., 2003). In general, myostatin exists in an inactive state with the part of its mature C-terminus non-covalently bound to the N-terminal propeptide. This potential complex can be activated *in vitro* through the cleavage of the propeptide and the bone morphogenetic protein-1/tolloid (BMP-1/TLD) metalloprotease family members, suggesting that BMP-1/TLD proteases play a critical role in the activation of myostatin *in vivo* (Lee, 2008). Mutation of the C-terminal polypeptide leads to muscle overgrowth in cattle and mice, indicating that myostatin is located in the C-terminal region of the propeptides (Wolfman et al., 2003). Interestingly, full-length myostatin may have some activity associated with low-affinity binding to its receptor and may produce contradictory effects. Incubation of isolated muscle cells from young rats, C2C12 and neonatal cardiomyocytes with *E. coli*-derived full-length myostatin results in increased proteolysis of oxidized soleus associated with increases in atrogin-1, MuRF-1 and LC3 concentrations; the extensor digitorum longus shows decreased protein turnover and increases only in atrogin-1 expression, similar to the findings for C2C12 cells (Manfredi et al., 2017). In addition, cardiomyocytes show a decrease in synthesis rate and an increase in proteolysis (Manfredi et al., 2017). When the growth differentiation factor 11 (GDF11) propeptide-Fc, an inhibitor of GDF11/myostatin, is injected into the hind limbs of C57BL/6J mice, the mass of skeletal muscle and the cross-sectional area of muscle fibers is increased and skeletal muscle hypertrophy is induced in adult mice (Jin et al., 2019). Conversely, no difference in protein synthesis is observed when full-length myostatin is overexpressed in neonatal cardiomyocytes (Morissette et al., 2006). It is uncertain whether these differences are due to cell overexpression or exogenous myostatin stimulation.

The GDF11 is a member of the TGF-β superfamily that is closely related to myostatin. It is generally considered to have effects that are similar to those of myostatin. Like other TGFβ family members, GDF11 and myostatin precursor proteins are proteolytically processed to form biologically-active carboxy-terminal dimers and their active domains share 90% amino acid sequence identity. Both GDF11 and myostatin predominantly utilize the type II activin receptor kinases II-A and II-B (ActRIIB) and the type I activin receptor-like kinases 4 and 5 (ALK4/5) to elicit signal transduction *via* Smad 2 and 3 (Lee and McPherron, 2001; Rebbapragada et al., 2003; Andersson et al., 2006). Binding of myostatin to ActRIIB is inhibited by the activin-binding protein follistatin and, at higher concentrations, by the myostatin propeptide (Lee and McPherron, 2001). Signaling of GDF11 and myostatin is fully regulated by extracellular binding proteins, including follistatin, follistatin-like 3 (FSTL3), decorin, and growth/differentiation factor associated serum proteins 1 and 2 (Sidis et al., 2006). Despite their similarities in protein sequence, receptor utilization, and signaling, accumulating evidence suggests that GDF11 and myostatin have distinct functions in many situations. Myostatin negatively regulates skeletal muscle development and cardiac muscle mass and modulates metabolic processes, whereas GDF11 is mainly involved in mammalian tissue aging, bone growth, and nerve and myocardial development (Walker et al., 2016). For example, endogenous GDF11 is a negative regulator of hippocampal neurogenesis in adult mice (Mayweather et al., 2021). GDF11 protects against diabetic cardiomyopathy by regulating the SIRT1 signaling pathway (Zhu et al., 2021). However, the functions of myostatin may not be limited to skeletal muscle, but may additionally influence other tissues including cardiac muscle, adipocytes, bone, and brain (Rodgers and Garikipati, 2008). More notably, there may be mutual regulation of myostatin and GDF11 functions. Recent findings show that deletion of myostatin up-regulates expression of GDF11, activates the bone morphogenetic protein (BMP) signal pathway, promotes maturation of differentiated cells and chondrocytes, inhibits formation of osteoclasts, and promotes osteogenic differentiation and osteogenesis (Suh et al., 2020).

## Myostatin Signaling Pathway in Skeletal Muscle

### Myostatin Signaling Pathway and its Target Genes

**FIGURE 1:**
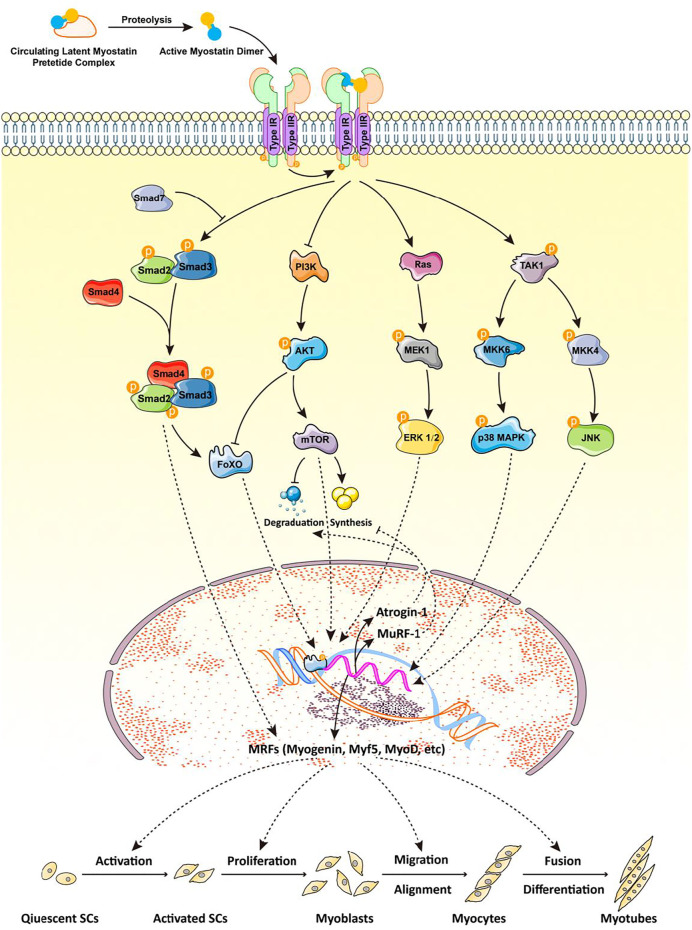
Myostatin and its signaling pathway are involved in myogenesis. The myostatin dimer first binds to ActRIIB, then to ALK4/5 to form a complex; Smad2/3/4 enters the nucleus to regulate the expression of target genes. Different transcription factors bind to the Smad2/3/4 complex, resulting in various functions of the Smad signaling pathway. Smad7 can prevent the phosphorylation of R-Smad by ActRI, thus blocking the myostatin signaling pathway mediated by Smad. The nonclassical pathway of myostatin signaling involves the PI3K/Akt/mTOR and MAPK signaling pathways, the latter of which mainly includes ERKs, JNKs, and p38MAPK. All of these pathways mediate the transcription of myogenic regulatory factors (myogenin, Myf5, MyoD), MuRF-1, and atrogin-1 to regulate myogenic differentiation and skeletal muscle quality. ActRIIB: activin receptor kinases II-B; ALK4/5: activin receptor-like kinase 4/5; R-Smad: receptor-regulated Smad; PI3K: phosphoinositide 3-kinase; Akt: protein kinase B; mTOR: mammalian target of rapamycin; MAPK: mitogen-activated protein kinase; ERK: extracellular signal-regulated kinase; JNK: c-Jun N-terminal kinase; Myf5: myogenic factor 5; MyoD: myoblast determination protein 1; MuRF-1: muscle RING-finger protein-1.

Myostatin is a secretory protein that transmits its signal to the nucleus through a series of tandem reactions. The myostatin dimer first binds to ActRIIB, then forms a complex with ALK4/5; the Smad2/3/4 complex enters the nucleus to regulate the expression of target genes (Figure 1). Various transcription factors bind to Smad2/3/4, resulting in different functions of the Smad signaling pathway. Among these factors, cytoplasmic proteins SnoN and Ski bind to Smad2/3/4 to block the activity of the Smad complex, then recruit co-repressor and histone acetylase complex to inhibit the expression of target genes. In addition, an *in vivo* study of muscle function in *Smad7* ^−/−^ mice demonstrated that Smad7 can prevent the phosphorylation of R-Smad proteins by ActRI and degrade the TGF-β receptor, thus blocking Smad-mediated myostatin signaling (Cohen et al., 2015).

Myostatin signaling can also be independent of the expression of genes regulated by Smad. For example, mitogen-activated protein kinases (MAPKs) including extracellular signal-regulated kinase (ERKs), c-Jun N-terminal kinases (JNKs), and p38 mitogen-activated protein kinases (p38MAPKs) are involved in the signal transduction pathway of myostatin (Huang et al., 2007; Gui et al., 2012) (Figure 1). In C2C12 cells, myostatin activates p38MAPK through the TAK1-MKK6 pathway and inhibits proliferation of myoblasts (Philip et al., 2005); it also induces the activation of JNK by binding to the myostatin receptor, ActRIIB, further inhibiting the proliferation and differentiation of myoblasts (Huang et al., 2007). *In vitro*, myostatin also reduces Akt/TORC1/p70S6K signaling, inhibiting myoblast differentiation and myotube size (Trendelenburg et al., 2009). Using siRNA to decrease expression of regulatory-associated protein of mTOR (RAPTOR), a component of TORC1, blocks the Akt/mTOR pathway, increases myostatin-induced phosphorylation of Smad2, and establishes a signal-amplification role for myostatin in the blockade of Akt (Trendelenburg et al., 2009). This suggests that inhibition of myogenic differentiation by myostatin is mediated at least in part by perturbation of Akt/TORC1 signaling. *In vivo*, FSTL3 is a downstream regulator of ActRII signal transduction and its expression is regulated by ActRII, activin A, myostatin, and GDF11. In addition, the E3 ubiquitin ligase, Smurf1, is a key downstream effector of the ActRII signal mediated by activin and can increase the proteasome-dependent degradation of sarcoplasmic reticulum Ca^2+^ ATPase (Roh et al., 2019). In conclusion, myostatin may regulate the expression of its downstream target genes through many Smad protein-independent signal transduction pathways to produce a variety of biological functions (Figure 1).

### Myostatin and the BMP Signaling Pathway

The BMP family is composed of numerous proteins and belongs to the TGF-β superfamily, together with myostatin (Chen et al., 2004). BMPs function in most tissues, and cell type-specific output of BMP signaling is essential for proper tissue function and differentiation. BMP has been widely studied because of its role in the regulation of bone development; however, its potential role in muscle growth has also been gradually investigated. BMP signaling is necessary for skeletal muscle satellite cell proliferation and its down-regulation triggers cell cycle exit and differentiation *in vivo* (Friedrichs et al., 2011; Ono et al., 2011). Notably, myostatin deficiency also has a positive effect on bone formation (Tang et al., 2020). The ability of TGF-β superfamily members, including BMP2, BMP4, BMP7, and TGF-β, to regulate mesenchymal cell differentiation has been firmly established (Hoffmann and Gross, 2001). For instance, BMP2 and BMP7 can promote adipogenesis, osteogenesis, and chondrogenesis. Crosstalk between BMP and myostatin has also been identified. For example, myostatin specifically antagonizes BMP7 by competing for binding to ActRIIB to regulate adipogenesis (Rebbapragada et al., 2003). In addition, both BMP and myostatin can affect the PI3K/Akt/mTOR pathway (Rocher and Singla, 2013) and their effects on muscle mass require transcription factors Smad2/3 and Smad1/5/8 (Jen et al., 1992; Miyazono and Miyazawa, 2002; Sartori et al., 2013a), respectively. Accumulating evidence shows that myostatin and BMP have opposing effects on skeletal muscle. *MSTN* ^−/−^ mice exhibit hypertrophy and hyperplasia, and inhibition of BMP signaling reverts the hypertrophic phenotype but not the hyperplasia of skeletal muscle (Sartori et al., 2013b). Mechanistically, lower concentrations of phosphorylated Smad2/3 lead to the release of Smad4, which is recruited into the BMP signaling pathway to promote hypertrophy and counteract atrophy (Sartori et al., 2013b). Conversely, more Smad4 is available for phosphorylated Smad2/3 when the BMP pathway is blocked or myostatin expression is increased, resulting in atrophy (Sartori et al., 2013b). This indicates that muscle hypertrophy caused by myostatin inhibition results from activation of the BMP pathway, further suggesting that BMP signaling plays an important role in controlling muscle maintenance, growth, and atrophy. However, BMP is a multifunctional growth factor, which is different from myostatin. In short, BMP regulates not only muscle development and bone growth but also embryonic axis formation (Bier and Robertis, 2015), prostatic hyperplasia (Liu et al., 2021), breast cancer cell proliferation (Sharma et al., 2021), cranial bone development (Chen et al., 2020), and neurovascular homeostasis (Petersen et al., 2021).

### Self-Regulation Mechanism of Myostatin

In addition to regulating a large number of downstream target genes, myostatin auto-regulates its promoter through negative feedback inhibition. Myostatin can induce suppression of Smad7 expression, while the overexpression of Smad7 in turn inhibits the activity of the myostatin promoter (Forbes et al., 2006). In addition, interference with its signaling can prevent myostatin from inducing Smad7 promoter activity. In myotubes expressing non-functional myostatin, the expression of Smad7 mRNA decreases, while the expression of myostatin increases (Forbes et al., 2006). These observations indicate that myostatin automatically regulates its gene expression through a Smad7-dependent mechanism in muscle-derived cells. In eukaryotes, transcriptional regulation is not the only mechanism through which gene expression is controlled. For example, overexpression of miR-499 leads to reduced myostatin 3′-UTR activity (Bell et al., 2010), and the overexpression of miR-208a can reduce the expression of myostatin (Callis et al., 2009). Interestingly, a naturally occurring gain-of-function mutation in the 3′-UTR region of the Texel sheep myostatin gene creates a miR-206 site that causes translational inhibition of myostatin expression (Clop et al., 2006). *Via* Smad3, myostatin also up-regulates the expression of miR-27a/b, which in turn targets and represses myostatin, forming a novel miRNA-mediated negative myostatin auto-regulatory loop during myogenesis (McFarlane et al., 2014).

## Myostatin Regulates Skeletal Muscle Growth and Development

### Myostatin Regulates the Proliferation and Differentiation of Skeletal Muscle Satellite Cells

Aside from being a myogenic regulatory factor, myostatin is also involved in myogenic differentiation. The majority of studies have demonstrated that myostatin negatively regulates the proliferation (Thomas et al., 2000b; Taylor et al., 2001) and differentiation (Langley et al., 2002) of skeletal muscle satellite cells. Myostatin negatively regulates the G1/S phase transition of the cell cycle by specifically up-regulating cyclin-dependent kinase inhibitor p21WAF1/CIP1 and reducing the level and activity of cyclin-dependent kinase 2 in myoblasts (Thomas et al., 2000a; Joulia et al., 2003; McCroskery et al., 2003), resulting in the arrest of myoblasts in the G1 phase of the cell cycle to maintain the static state of satellite cells. In addition, endogenous myostatin protects undifferentiated myoblasts from apoptosis, and its overexpression inhibits the withdrawal of myoblasts in the G0/G1 phase from the cell cycle and increases the aggregation of cells in the G2 phase. Conversely, over-expression of myostatin inhibits the protein levels of MyoD and myogenin and inhibits the activation of myoblast differentiation (Joulia et al., 2003). Importantly, *MSTN* is a downstream target gene of MyoD, which may trigger myoblasts to exit the cell cycle by regulating the expression of myostatin (Spiller et al., 2002). Similarly, myostatin has concentration-dependent effects on the proliferation and activity of C2C12 cells because it inhibits their proliferation at high concentrations (80–400 nM) (Taylor et al., 2001; Langley et al., 2002) and promotes their proliferation at low concentrations (2–20 nM) (Rodgers et al., 2014). This may result from the inhibition of endogenous myostatin expression, thereby stimulating proliferation and differentiation and increasing the apoptosis rate of myoblasts. Also, miR-27b can target *MSTN* to regulate the proliferation of sheep skeletal muscle satellite cells (Zhang et al., 2018). Notably, myostatin decreases the diameter of differentiated myotubes (Trendelenburg et al., 2009). Recently, a study on the downstream target of myostatin, *Smad2*, showed that the knock-out of *Smad2* expression in primary myoblasts does not affect the efficiency of myogenic differentiation but produces smaller myotubes with reduced expression of the terminal differentiation marker, myogenin; conversely, overexpression of Smad2 stimulates the expression of myogenin and enhances cell differentiation and fusion (Lamarche et al., 2021).

### Role of Myostatin in Transformation of Skeletal Muscle Fiber Types

In mammals, the myotubes resulting from the proliferation and differentiation of skeletal muscle satellite cells form single muscle fibers that eventually form muscle fiber bundles. Skeletal muscle consists of different types of muscle fibers. Depending on the proportion of myosin heavy chain (MyHC) subtypes, muscle fibers can be divided into slow oxidation (type I), fast oxidation (type II A), intermediate (type II X), and fast glycolysis (type II B) muscle fibers. After most mammals are born, the number of muscle fibers is essentially constant but there is a continuous transformation between fiber types (Schiaffino and Reggiani, 2011). External factors, such as exercise, nutrition, and stress can directly affect the transformation of muscle fiber types (Jia et al., 2014).

The mRNA expression of MyHC-I in skeletal muscle of *MSTN* knockout mice is down-regulated, whereas that of MyHC-IIB is up-regulated; moreover, the proportion of type I muscle fibers is decreased, whereas that of type II muscle fibers is increased. In addition, fast muscle fibers are the main cause of muscle hypertrophy in mice (Girgenrath et al., 2005; Hennebry et al., 2009). Proteomic analysis of Belgian blue cattle skeletal muscle with natural mutation of the myostatin gene found that proteins associated with the fast muscle fiber phenotype are up-regulated (Bouley et al., 2005). It can be concluded that *MSTN* knockout may lead to proliferation of fast glycolysis muscle fibers. However, the distribution of this muscle fiber type is variable, and inhibition of myostatin by a neutralizing antibody in mature individuals will not change the muscle fiber type to favor fast glycolysis (Girgenrath et al., 2005). Recent studies have revealed that miRNAs and lncRNAs are also involved in regulating the transformation of muscle fiber types. For example, miR-182 is highly expressed in fast muscle, and miR-182-knockout mice show muscle loss and a change from a fast muscle to slow muscle fiber type compared with wild type animals (Zhang et al., 2016). Long intergenic non-coding RNA (linc-MYH) in the nucleus of fast muscle fibers inhibits the expression of the slow muscle gene and promotes the expression of the fast muscle gene (Sakakibara et al., 2014). Interestingly, dysregulation of miR-30e expression in *MSTN* ^−/−^ mice leads to changes in the composition of fiber types (Jia et al., 2017). In summary, non-coding RNA such as miRNA regulates the transformation of muscle fiber types controlled by the myostatin gene.

### Roles of Myostatin in Protein Synthesis and Degradation of Skeletal Muscle

Muscle growth and development are dynamic processes involving skeletal muscle protein synthesis and degradation. When the rate of protein synthesis exceeds that of degradation, the muscle shows growth, otherwise it shows atrophy. Protein synthesis is controlled by the efficiency of mRNA translation and the tissue concentration of translational machinery (Chaillou et al., 2014; Figueiredo and McCarthy, 2019). Protein degradation is mainly carried out *via* ubiquitin proteasomal, autophagic/lysosomal, and calpain-dependent pathways (Sandri, 2013). Accumulating studies have shown that including Wnt/β-catenin signaling pathway, Hippo signaling pathway, mTORC1 and c-myelocytomatosis oncogene (c-Myc) mediated ribosomal biogenesis, IGF-1/Akt, MAPK/ERK and NF-κB signal pathway mediated translation efficiency are extensively involved in skeletal muscle protein synthesis and degradation (Watt et al., 2015; Kirby, 2019; Vainshtein and Sandri, 2020). Excellent reviews may be consulted for more information on the mechanisms of protein synthesis and degradation (Mirzoev, 2020; Vainshtein and Sandri, 2020). MuRF-1 and atrogin-1 are critical muscle-specific E3 ligases that mediate muscle atrophy (Liu et al., 2019). As mentioned above, compared with untreated muscles, those incubated with full-length myostatin exhibit an increase in proteolysis mediated by atrogin-1 and MuRF-1, suggesting that myostatin may inhibit protein synthesis and/or increase protein degradation in muscles (Manfredi et al., 2017). Notably, this regulation was found to be FoxO-dependent (McFarlane et al., 2006). The up-regulation of atrogin-1, MuRF-1, and several autophagy-related genes is normally blocked by Akt *via* negative regulation of FoxO transcription factors (Bonaldo and Sandri, 2013). Myostatin treatment blocks the IGF1/PI3K/Akt pathway and activates FoxO1, leading to increased expression of atrogin-1 (Bonaldo and Sandri, 2013). Recent *in vitro* and *in vivo* studies have dissected the role of the myostatin signaling pathway on muscle protein degradation and shown that Smad2 and Smad3 are transcription factors mediating the effect of myostatin on muscle mass (Sartori et al., 2009). Further studies assessed the inhibitory effect of myostatin/Smad2/3 signaling on the IGF1/Akt/mTOR pathway, suggesting that myostatin, FoxOs, and Smads synergistically affect muscle protein synthesis and degradation, thereby regulating muscle mass (Amirouche et al., 2009; Sartori et al., 2009).

### Myostatin Trigger ROS Generation and Oxidative Stress in Skeletal Muscle

Skeletal muscle fibers contain a large number of mitochondria, which produce ATP *via* oxidative phosphorylation and provide energy for muscle contraction. In the process, mitochondria also produce several types of “reactive species” as side product, such as ROS and reactive nitrogen species (RNS). In fact, mitochondria have been proved to play a key role in the treatment of skeletal muscle ROS. As mentioned above, myostatin is involved in regulating the transformation of skeletal muscle fiber types. Compared with wild-type mice, muscle hypertrophy is observed with a higher proportion of glycolytic-to-oxidative myofibers in myostatin knockout mice (Hennebry et al., 2009), suggesting that myostatin may affect the mitochondrial content of skeletal muscle. *MSTN* ^−/−^ mice also have less skeletal muscle mitochondrial DNA and a lower mitochondrial volume compared with wild-type mice (Ploquin et al., 2012). However, the mechanism leading to these mitochondrial defects remains to be established. Myostatin promotes mitochondrial biogenesis *via* Smad signaling (Ge et al., 2012). Notably, myostatin also stimulates mitochondrial division by regulating the expression of Drp1 and Fis1 (Manfredi et al., 2019), which indicates that mitochondrial circulation is enhanced. However, the physiological significance of this stimulatory function is currently unclear.

**FIGURE 2:**
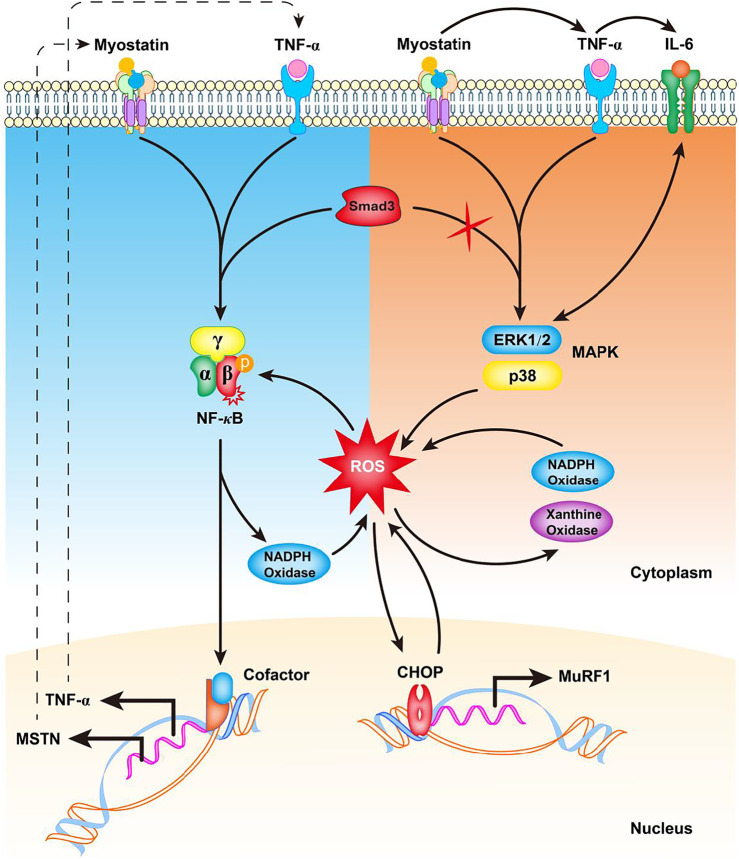
Potential mechanism of myostatin-induced ROS generation in skeletal muscle. In the presence of Smad3, increased myostatin induces TNF-α production *via* NF-κB signaling, increasing the production of ROS by NADPH oxidase. The induced ROS cause a feed forward loop, further increasing myostatin levels *via* NF-κB signaling of TNF-α. In the absence of Smad3, myostatin induces TNF-α and IL-6 to activate p38 and ERK MAPKs to promote Nox- and XO-mediated ROS generation. The induced ROS result in increased C/EBP homologous protein (CHOP) levels and up-regulation of MuRF-1 transcription. An increased CHOP protein level in turn induces ROS production that further leads to increased myostatin production. TNF-α: tumor necrosis factor alpha; ROS: reactive oxygen species; NF-κB: nuclear factor-κB; IL-6: interleukin 6; Nox: NADPH oxidase; XO: xanthine oxidase; C/EBP: CCAAT/enhancer-binding protein.

Although the production of ROS is inevitable, especially during skeletal muscle contraction and physical exercise, the body has an adaptive defense system involving a large number of antioxidants, such as superoxide dismutase, glutathione peroxidase, and catalase that is essential to balance ROS content in muscle (Jiang et al., 2020). Accumulating evidence suggests that myostatin is a pro-oxidant and signals the generation of ROS in skeletal muscle. Myostatin treatment results in a marked increase of ROS concentration in C2C12 myotubes (Aravena et al., 2020); however, the underlying mechanism remains unclear. Myostatin can trigger the production of second messenger ROS mediated by canonical Smad2/3, NF-κB, TNF-α, and NADPH oxidase signaling to target muscle-specific E3 ligases MuRF-1 and atrogin1 in skeletal muscle (Sriram et al., 2011) (Figure 2). Elevated TNF-α in turn stimulates the expression of myostatin, which can cause muscle atrophy by activating proteasome-mediated intracellular protein catabolism (Sriram et al., 2011) (Figure 2), suggesting that inhibiting myostatin-triggered ROS can reduce muscle wasting associated with sarcopenia. In addition, mice lacking Smad3, the downstream signaling molecule of TGF-β and myostatin, exhibit skeletal muscle atrophy because of elevated myostatin levels. Investigation of Smad3-independent mechanisms by which myostatin induces muscle atrophy in *Smad3* ^−/−^ muscle revealed that Smad3 is necessary for myostatin to induce NF-κB (p65)-mediated ROS formation (Sriram et al., 2014) (Figure 2). Further studies demonstrated that the production of ROS is initiated through MAPK (p38 and ERK) signaling pathways, which finally trigger oxidative stress-dependent muscle atrophy (Sriram et al., 2014) (Figure 2).

## Concluding Remarks

Myostatin, as the only known muscle growth inhibitor, plays a key role in muscle cell proliferation and differentiation, muscle fiber type transformation, muscle physiology, and muscle protein synthesis and degradation. With the interaction mechanism of the myostatin, GDF11, and BMP signaling pathways having been revealed, the role of myostatin in fat metabolism and bone development has gradually become a new area of intense research. Considering that myostatin can coordinate the regulation of skeletal muscle development in many ways, it is anticipated that an in-depth study of myostatin’s signaling mechanism and regulatory network will further reveal its role in protein synthesis and degradation, fat metabolism, and bone development.
